# Supplementary material for: Enhancing Plant Resistance to Sri Lankan Cassava Mosaic Virus Using Salicylic Acid
Source: Metabolites. 2025 Apr 10;15(4):261. doi: 10.3390/metabo15040261 (PMC12029932; doi:10.3390/metabo15040261)
Supplement: Supplementary file 1 [file metabolites-15-00261-s001.zip › Figure S2.pdf]

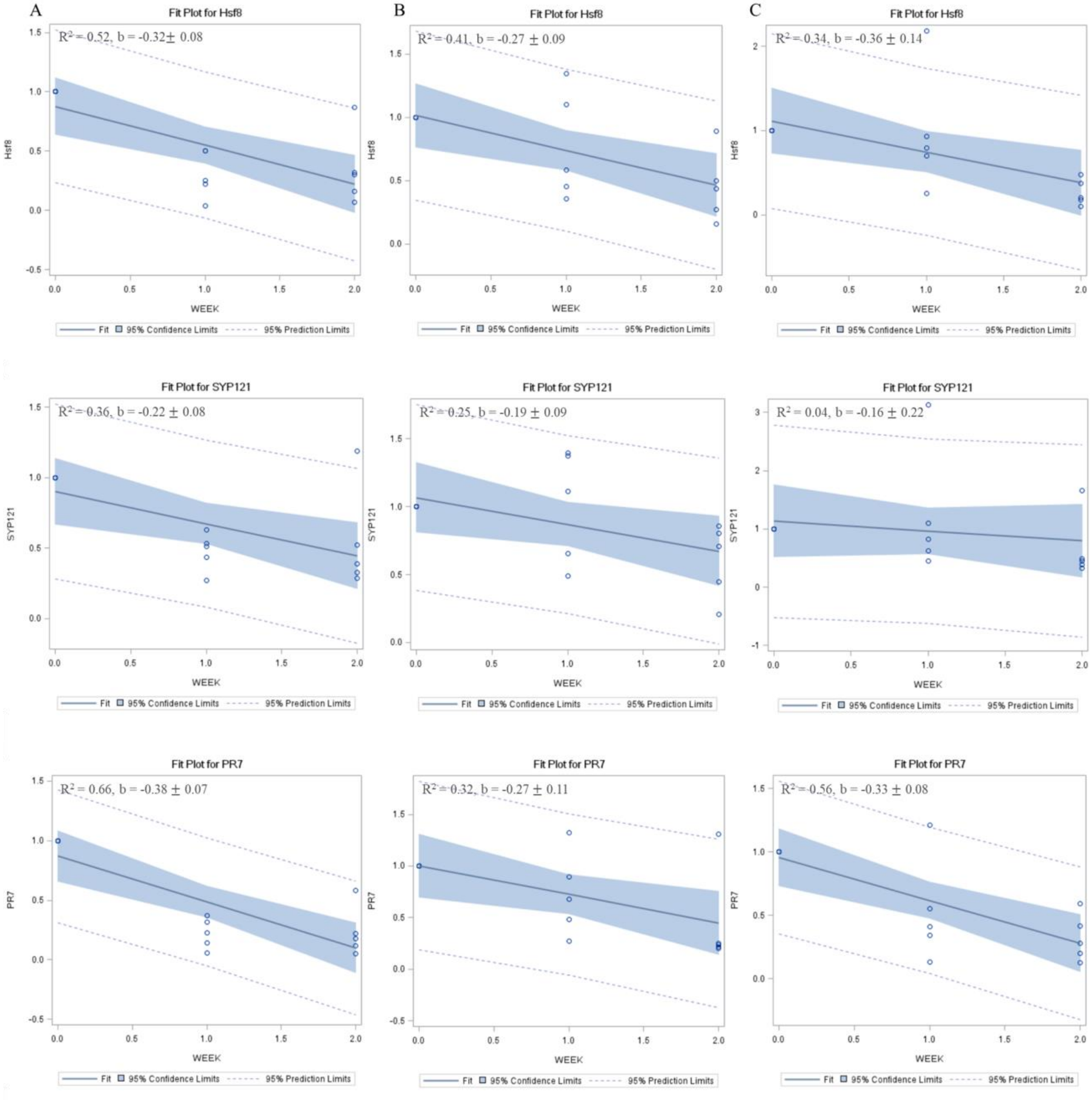

Figure S2 Alterations in the expression profiles of *Hsf8*, *SYP121*, and *PR7* genes at 0, 1, 2, 3, and 4 weeks post-treatment with SA, as determined by regression analysis. The results are presented as b ± SE. The cassava plants were infected with SLCMV after spraying with SA at concentrations of 0 mg/mL (A), 100 mg/mL (B), or 200 mg/mL (C).
