# Supplementary material for: Enhancing Plant Resistance to Sri Lankan Cassava Mosaic Virus Using Salicylic Acid
Source: Metabolites. 2025 Apr 10;15(4):261. doi: 10.3390/metabo15040261 (PMC12029932; doi:10.3390/metabo15040261)
Supplement: Supplementary file 1 [file metabolites-15-00261-s001.zip › Table S1.pdf]

Additional Data

Enhancing plant resistance to Sri Lankan cassava mosaic virus using salicylic acid

Table S1 List of primers used for RT-qPCR

No	Gene name	Primer Name	Primer Sequence (5' →3')	References
1	*PR7*	MePR-7f5F	CAG TCC CCA AGA AAT GGA GA	Irigoyen *et al.* (2020)
2	*PR7*	MePR-7f5R	TCC TGA ACG GTG TTT GAT GA	Irigoyen *et al.* (2020)
3	*PR9*	MePR-9eF	GGA GGC CAT GCT TAC TGA TC	Irigoyen *et al.* (2020)
4	*PR9*	MePR-9eR	CTC CAT AGT AGG AGT AGT GTC	Irigoyen *et al.* (2020)
5	*HSP90.9*	QMeHSP90.9F	CGC TAT ATA TGC TCC GCA AA	Wei *et al.* (2021)
6	*HSP90.9*	QMeHSP90.9R	TGA GAT GAG AGA TAA AAG GCA CA	Wei *et al.* (2021)
7	*Hsf8*	QMeHsf8F	TGA AGA AAA TTC ATA GAC GGA AGC	Wei *et al.* (2021)
8	*Hsf8*	QMeHsf8R	CGT GGA TAT TTT CAT TTT GCA C	Wei *et al.* (2021)
9	*SRS1*	QMeSRS1F	CCA CCA ACT GAA ATC CCA CA	Wei *et al.* (2021)
10	*SRS1*	QMeSRS1R	CTC CTG CAA CAA GTC CTA CAC C	Wei *et al.* (2021)
11	*SYP121*	MeSYP121F	ATC CAG ATG TCA GAG ATG CC	Yoosomboon *et al.* (2021)
12	*SYP121*	MeSYP121R	GAG CCT CCA AAC GAA CCT TA	Yoosomboon *et al.* (2021)
13	*PR1*	MePR1F	GCA AAG CAG AAC ACT CAT TTC CT	Yoodee *et al.* (2018)
14	*PR1*	MePR1R	CGA GCA CAC CCA ATT CTC CT	Yoodee *et al.* (2018)
15	*WRKY59*	WRKY59F	GCT CCA ACA CAA AGA AGA AGC C	Yoodee *et al.* (2018)
16	*WRKY59*	WRKY59R	CAA CTA CCC TCC GCA CAC AT	Yoodee *et al.* (2018)

No	Gene name	Primer Name	Primer Sequence (5' →3')	References
17	*PDF2*	PDF2.2F	TTC CTC CTC TTG CTG CTT GT	Yoodee *et al.* (2018)
18	*PDF2*	PDF2.2R	CTC ACG CAT ACA CCC TTG AA	Yoodee *et al.* (2018)
19	*UBQ10*	MeUBQ_F	CGA CTA CAA CAT CCA GAA GG	Irigoyen *et al.* (2020)
20	*UBQ10*	MeUBQ_R	TTG TGT CGG AAC TCT CCA CC	Irigoyen *et al.* (2020)
